# The Effect of Different Cleaning Protocols of Polymer-Based Prosthetic Materials on the Behavior of Human Gingival Fibroblasts

**DOI:** 10.3390/ijerph17217753

**Published:** 2020-10-23

**Authors:** Vygandas Rutkunas, Rokas Borusevicius, Dominyka Liaudanskaite, Urte Jasinskyte, Saulius Drukteinis, Virginija Bukelskiene, Eitan Mijiritsky

**Affiliations:** 1 Institute of Odontology, Faculty of Medicine, Vilnius University, 03101 Vilnius, Lithuania; rokas.borusevicius@gmail.com (R.B.); dominyka.liaudanskaite@yahoo.com (D.L.); saulius.drukteinis@gmail.com (S.D.); 2 Institute of Biochemistry, Life Sciences Center, Vilnius University, 03101 Vilnius, Lithuania; u.jasinskyte@gmail.com (U.J.); virginija.bukelskiene@gmail.com (V.B.); 3 Head and Neck and Maxillofacial Surgery, Department of Otolaryngology, Tel-Aviv Sourasky Medical Center, Sackler Faculty of Medicine, Tel-Aviv 6997801, Israel; mijiritsky@bezeqint.net; 4 The Maurice and Gabriela Goldschleger School of Dental Medicine, Tel-Aviv University, Tel-Aviv 6997801, Israel

**Keywords:** polymers, surface, roughness, contact angle, fibroblasts, proliferation, PMMA, PEEK, PEKK, cleaning

## Abstract

Dental implant abutment and prosthetic materials, their surface treatment, and cleaning modalities are important factors for the formation of a peri-implant soft tissue seal and long-term stability of bone around the implant. This study aimed to investigate the influence of a polymeric material surface cleaning method on the surface roughness, water contact angle, and human gingival fibroblasts (HGF) proliferation. Polymeric materials tested: two types of milled polymethylmethacrylate (PMMA-Ker and PMMA-Bre), three-dimensionally (3D) printed polymethylmethacrylate (PMMA-3D), polyetheretherketone (PEEK), and polyetherketoneketone (PEKK). Titanium (Ti) and zirconia oxide ceramics (ZrO-HT) were used as positive controls. A conventional surface cleaning protocol (CCP) was compared to a multi-step research cleaning method (RCP). Application of the RCP method allowed to reduce S_a_ values in all groups from 0.14–0.28 µm to 0.08–0.17 µm (*p* < 0.05 in PMMA-Ker and PEEK groups). Moreover, the water contact angle increased in all groups from 74–91° to 83–101° (*p* < 0.05 in the PEKK group), except ZrO-HT—it was reduced from 98.7 ± 4.5° to 69.9 ± 6.4° (*p* < 0.05). CCP resulted in higher variability of HGF viability after 48 and 72 h. RCP application led to higher HGF viability in PMMA-3D and PEKK groups after 48 h, but lower for the PMMA-Ker group (*p* < 0.05). After 72 h, no significant differences in HGF viability between both cleaning methods were observed. It can be concluded that the cleaning method of the polymeric materials affected surface roughness, contact angle, and HGF viability at 48 h.

## 1. Introduction

Dental implants have become a highly widespread and predictable treatment method for rehabilitating partially or completely edentulous patients. The soft tissues surrounding dental implants play a significant role in ensuring esthetics, preventing the invasion of the microorganisms and other hazardous agents, and avoiding crestal bone loss, thus ensuring the longevity of implant-supported restorations [1,2,3,4,5]. The quantity and quality of soft tissue are essential for bone stability around dental implants [6,7]. 

The seal of soft tissue around the implant abutment and prosthesis shields the underlying tissues from the environment of the oral cavity. When this barrier of soft tissues is damaged, there is a risk that microorganisms can reach the surface of the dental implant [1,8]. Inflammation in this area might eventually lead to bone loss, compromised aesthetics, and peri-implantitis [2,9,10]. 

The usage of titanium base abutments became a standard in implant prosthodontics. When placed intraorally, only a small part of this abutment and predominantly selected prosthetic material is in contact with peri-implant tissues. A wide variety of prosthetic materials can be used in these situations: metal alloys, ceramics, and polymer-based materials. For simplicity, further in text implant abutment and prosthetic materials will be referred to as “abutment materials.” 

Soft tissue and bone response may be influenced by the type of implant abutment material and surface properties [1,2,11]. The relationship between the material and the resulting condition of soft tissue has been reported in multiple studies [11,12,13,14,15]. The majority of the studies have investigated the effects of titanium and zirconia materials [16,17]. However, only a few studies have been conducted on polymer-based materials, which can be used as temporary or permanent prosthetic materials. Concerns of lower biocompatibility of PMMA (polymethylmethacrylate) materials were raised in the literature (e.g., cytotoxicity of PMMA monomers, higher surface bacterial contamination compared to other materials); however, they are widely used for fabrication of provisional restorations [18,19,20,21,22]. PEEK (polyetheretherketone) materials have gained more attention in recent years as an alternative to titanium and ceramics [15,18,19,23,24]. Scientific evidence shows that PEEK and its modifications are favorable for fibroblast and epithelial cell response and might provide less biofilm formation [15,18,24]. On the other hand, studies regarding 3D printed materials and novel polymers (e.g., PEKK—polyetherketoneketone) remain limited.

Material surface microtopography can determine the quality of soft tissue integration around implant abutment [1]. The tendency of rough zirconia surfaces (Ra = 0.19 ± 0.03 μm) to provide higher fibroblast content after 3 and 24 h, but lower after 48 and 72 h compared to smooth surfaces (Ra = 0.05 ± 0.01 μm) was demonstrated by recent research [25]. On the other hand, surface roughness (Ra) higher than 0.2 μm tends to increase bacterial adhesion [26]. The formation of plaque and reproduction of bacteria depends on the characteristics of implant abutment surfaces and play an important role in the pathogenesis of infection [27,28,29]. 

The hydrophilicity of the abutment surface is another factor influencing peri-implant tissue health [30,31]. The material is considered hydrophilic if the contact angle of a water droplet on the surface is below 90 degrees and hydrophobic if it exceeds 90 degrees [32]. Studies have demonstrated that hydrophilic abutment surfaces can promote fibroblast adhesion and proliferation and improve the soft tissue attachment [30,31]. Most polymeric materials (e.g., PMMA and PEEK) tend to be less hydrophilic than zirconia, but the correlation between their surface roughness values and water contact angle is not evident [20].

The contamination of the material surface might affect cell growth [33]. Cleaning of the implant abutment surface is important not only for the decontamination but also might modify surface properties, and thus cellular response [19,34,35,36]. Various cleaning protocols have been suggested to improve surface biocompatibility and cell proliferation. It has been shown that ultrasonic cleaning decreases the amount of debris on implant abutments and supposedly promote soft tissue healing [35]. The cleaning protocol used in cell proliferation research studies often differs from that used in the dental laboratory [36]. Therefore, it is unknown if the results of these studies can be applied to clinical practice.

There is a lack of knowledge on the soft tissue response to polymeric materials that can be fabricated using CAD/CAM (computer-aided design and computer-aided manufacturing) or 3D printing. Moreover, it is unknown how different cleaning protocols can affect their properties. 

The purpose of this study was to compare the effect of two different surface cleaning protocols of selected polymeric dental materials in regards to the surface roughness, hydrophilicity, and proliferation of human gingival fibroblast (HGF) cells using titanium and zirconia as positive controls. The null hypothesis tested was that there are no significant differences in surface roughness, hydrophilicity, and HGF proliferation between surface cleaning protocols and types of polymeric materials.

## 2. Materials and Methods 

Five types of polymeric materials were included in this study: polymethylmethacrylate (PMMA-Ker), polymethylmethacrylate composite (PMMA-Bre), 3D printed polymethylmethacrylate (PMMA-3D), polyetheretherketone reinforced with ceramic filler (PEEK), and polyetherketoneketone reinforced with titanium dioxide (PEKK) (Table 1). Two positive control groups were used: titanium (Ti) and zirconium oxide ceramic (ZrO-HT). 

### 2.1. Preparation of the Specimens

Specimens from each group, except the Ti and PMMA-3D, were milled using a CAM unit (Vhf S1 Impression, vhf camfacture AG, Ammerbuch, Germany). Titanium specimens were milled using DATRON D5 (DATRON AG, Mühltal, Germany). PMMA-3D specimens were printed using stereolithography technology (Form 2 printer, Formlabs, Somerville, MA, USA), and post-processed according to the manufacturer’s guidelines. Final specimen parameters were: height—2 mm, diameter—5 mm. In total, 84 specimens were prepared (12 specimens per group). 

### 2.2. Surface Polishing 

Each side of the specimens was polished according to the manufacturer’s protocol (Table 2). Specimens were repolished following the same protocol before every separate experiment was used in the study.

### 2.3. Surface Cleaning

Specimens were randomly selected (www.randomlists.com) for each cleaning group. 

Conventional cleaning protocol (CCP): specimens were disinfected in “Perform 2%” (Schülke & Mayr GmbH, Norderstedt, Germany) solution for 10 min, then rinsed with tap water for 30 s, soaked in isopropyl alcohol (Isopropyl alcohol, ≥99.7%, Sigma-Aldrich, St. Louis, MI, USA) for 10 min and washed in an ultrasonic cleaner (42,000 vibration/sec, Carrera 2505 PEARL Cosinus Ultrasonic Cleaner, Aquarius Deutschland GmbH, Düsseldorf, Germany) with distilled water for 3 min. 

Research cleaning protocol (RCP): specimens were soaked in “Decon” solution (Decon Laboratories™ Decon 90™, Fisher Scientific, NH, USA) and put on a laboratory tumbling table (Mini-Tumbling Table WT17, 25 rpm, angle of inclination 5°/10°, Biometra GmbH, Göttingen, Germany) for 24 h, rinsed with tap water 20 times, then rinsed with distilled water 10 times and soaked in 70% ethanol for 24 h. 

After washing, specimens were air-dried at room temperature for 24 h. The same protocol was repeated after every specimen polishing session just before the next experiment.

### 2.4. Profilometry

Surface mean roughness (Sa) was measured using a 3D optical profiler system (PLμ 2300, Sensofar, Sensofar Group, Barcelona, Spain) with a confocal objective 50×/0.8 A with FOV 255 × 191 µm (Nikon Lu Plan, Nikon Metrology NV, Leuven, Belgium). Five specimens from every material group were selected randomly, and 3 images of surface areas (two areas were randomly chosen on a surface periphery and one in the center) on every selected specimen were made. The images were processed, and Sa values measured using Gwyddion Software (Czech Metrology Institute, Jihlava, Czech Republic).

### 2.5. Water Contact Angle Measurements

To evaluate surface hydrophilicity, the mean water contact angle was measured for each material group. After the surface cleaning samples were subsequently placed in a Krüss EasyDrop system (KRÜSS GmbH, Hamburg, Germany) and deionized water droplets (16 Ω, 2 μL) were placed on the samples, pictures were taken after 10 s and finally analyzed using Krüss software (KRÜSS GmbH, Hamburg, Germany). Two measurements (one on each side of the droplet) were obtained, and the mean value was calculated. The sample chamber temperature was kept constant at 21 °C using a LabTech H50-500 water chiller (LabTech Srl, Sorisole BG, Italy). Five specimen surfaces were randomly selected from each material group resulting in 5 measurements. 

### 2.6. Cell Culture

Primary human gingival fibroblast (HGF) mono-layered culture was used. These cells were derived from healthy patient undergoing periodontal surgery using the technique described in previous research (approval of the national Bioethics committee No 158200-16-860-369) [37]. The cells from 6 to 12 passages were used in this experiment. HGF were grown in an IDMEM (Iscove’s Modified Dulbecco’s Medium; Gibco, Thermo Fisher Scientific, Waltham, MA, USA) with 10% FCS (Fetal calf serum; Gibco, Thermo Fisher Scientific, Waltham, MA, USA) and antibiotics (penicillin, 100 VV/mL, and streptomycin, 100 μg/mL; Gibco, Thermo Fisher Scientific, Waltham, MA, USA) in 50 mL plastic flasks (Greiner, Greiner Bio-One GmbH, Frickenhausen, Germany). The experiments carried out using 96 well plates (Greiner, Greiner Bio-One GmbH, Frickenhausen, Germany). Cells were grown in the incubator (Heracell™ 150i, Thermo Fisher Scientific, Waltham, MA, USA) at 37 °C, 5% CO_2_, 95% H_2_O) and HGF were passaged 2 times per week.

### 2.7. The Assessment of Fibroblast Proliferation on Different Dental Materials

Before the proliferation experiment, all the specimens were treated with UV-C light (Sylvania G15W T8 lamps, Feilo Sylvania Group, Shanghai Feilo Acoustics Co., Budapest, Hungary) with a peak wavelength of 253.7 nm for 7 min at a distance of 12 cm, resulting in irradiance of around 3.49 mW/cm^2^.

For the evaluation of cell proliferation, a suspension of HGF (30 × 10^3^ cells/mL) was prepared and poured into the plate wells with the specimens, 200 μL into each. Three specimens of every dental material were used per each time point. The amount of the cells grown on the specimens and on the control plastic surface was registered at 24, 48 and 72 h. The experiment was repeated 3 times. 

Amount of living cells in the well was registered using MTT test. At every check point cell growth medium was carefully removed from the well and 100 μL of MTT (3-(4,5-dimetiltiazol-2-il)-2,5-diphenyltetrazolium bromide; Merck Chemicals, Merck KGaA, Darmstadt, Germany) (1 mg/mL prepared in phosphate-buffered saline) was poured into every well. After 1 h of incubation at 37 °C, 5% CO_2_) MTT was removed and the formed formazan crystals were dissolved in 100 μL ethyl alcohol (96%). Then, 50 μL of the developed solution was transferred to the clean wells. Later, the optical density (OD) of the solvent and specimens was measured at wavelength of 570 nm using spectral scanning microplate reader (Varioskan Flash, Thermo Scientific, Waltham, MA, USA). The difference between every specimen under observation and the mean of solvent OD was subsequently calculated. The obtained OD is proportional to the number of live cells on the observed specimen. To compare the assays, the OD, equal to the count of live cells grown on every specimen, was described in ratio with the negative control group (control). The negative control group was considered to be the OD, which described the number of live cells grown on a plate well surface.

Ratio OD of the specimen/OD of the control group at 24 h shows if the surface of a specific material inhibits the cell growth (the ratio is below 1) or, on the contrary, promotes cell proliferation (the ratio is higher than 1). A value of 1 is considered the cell growth on the control group surface at 24 h.

### 2.8. Statistical Analysis

Statistical analysis was performed using R i386 4.0.0 (Lucent Technologies, Auckland, New Zealand). The graphs were plotted using the ggplot2 plugin (Lucent Technologies, Auckland, New Zealand). Data normality was tested, and parametric methods were used in case of normal data distribution; otherwise, a non-parametric analysis was performed. In the case of parametric analysis, the equality of variances was tested using two variances F-test (two-sided) for two groups and Levene’s test for homogeneity of variance (center—mean) for more than two groups. In the case of unequal variances, parametric tests were adjusted accordingly. To compare the means of two groups two-tailed independent samples *t*-test was used as a parametric test and two-sample Wilcoxon test (two-tailed with a normal approximation with continuity correction) as a non-parametric option. In the case of parametric means comparison for more than two groups, one-way ANOVA and subsequent Tukey’s post hoc tests. For non-parametric multiple-group means comparison Kruskal–Wallis rank-sum test and pairwise comparisons using the Wilcoxon exact rank-sum test with *p* value adjustment using Benjamini and Hochberg method were performed. The statistical significance level was set at *p* < 0.05.

## 3. Results

### 3.1. Surface Roughness

**Figure 1:**
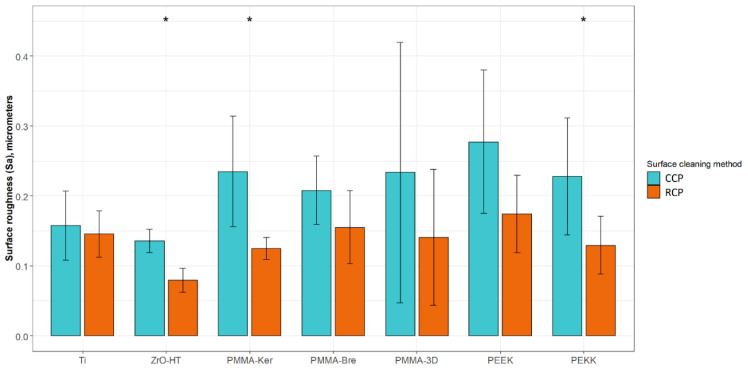
Sample surface roughness (Sa) values using two different cleaning protocols. The results are presented as averages +/− standard deviations. *—statistically significant differences (Wilcoxon, *p* < 0.05) comparing means of CCP and RCP for each material group. CCP—Conventional cleaning protocol; RCP—Research cleaning protocol.

**Figure 2:**
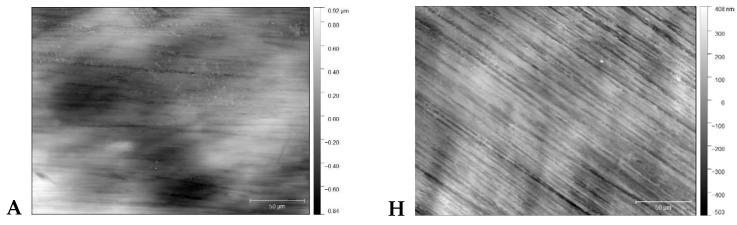

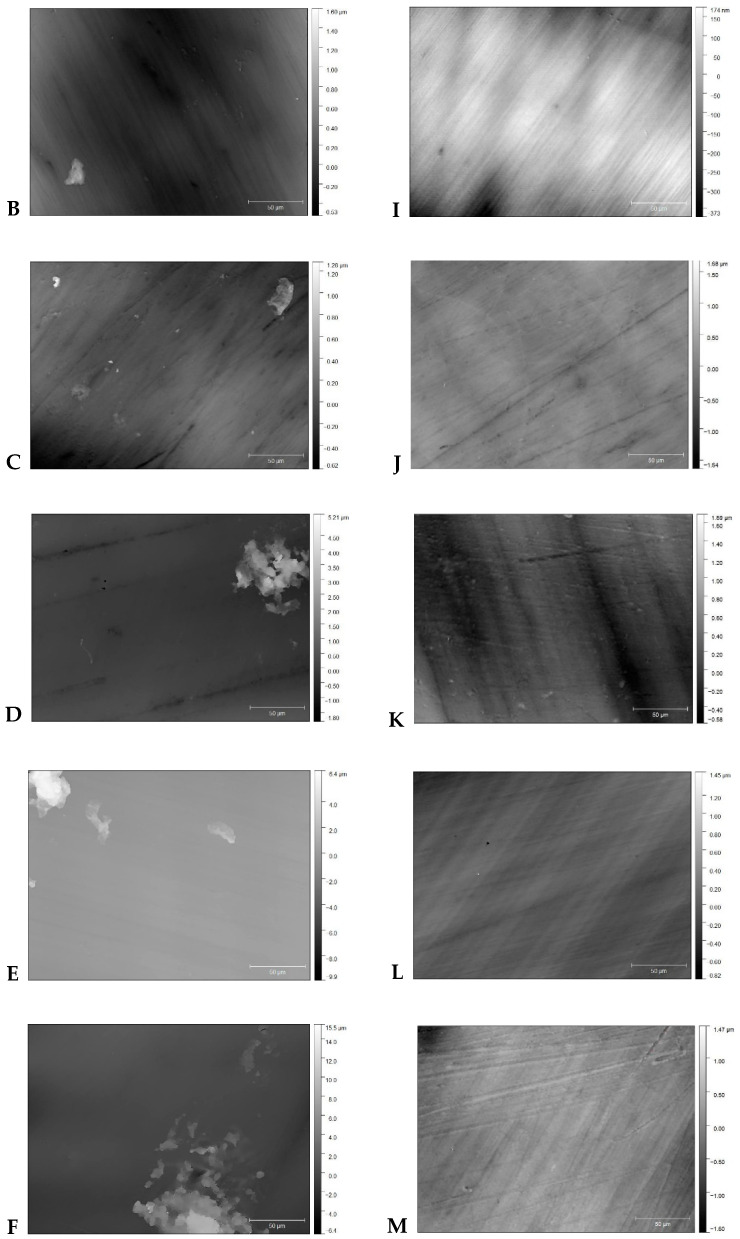

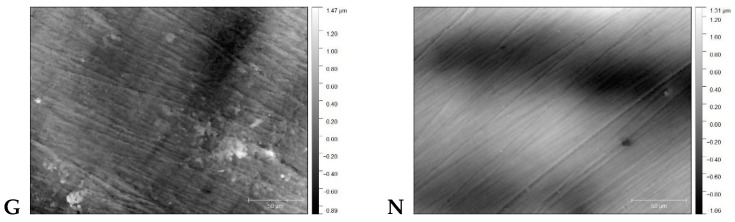
Material surface images after application of CCP (**A**—Ti, **B**—ZrO-HT, **C**—PMMA-Ker, **D**—PMMA-Bre, **E**—PMMA-3D, **F**—PEEK, **G**—PEKK) and RCP (**H, I, J, K, L, M, N** respectively) cleaning protocols obtained by confocal microscope (×50).

The results of surface profilometry are presented in Table 3. In the case of the CCP, no statistically significant differences between material groups were observed. After cleaning the specimens using the RCP, ZrO-HT group surface roughness was significantly lower (Kruskal–Wallis and pairwise Wilcoxon, *p* = 0.042) compared to Ti, PMMA-Ker, PMMA-Bre, PEEK, and PEKK groups. Significant surface roughness differences for each material group comparing two surface cleaning protocols are presented in Figure 1. Examples of material surfaces under a confocal microscope (PLμ 2300, Sensofar, Sensofar Group, Barcelona, Spain) are shown in Figure 2.

### 3.2. Contact Angle

**Figure 3:**
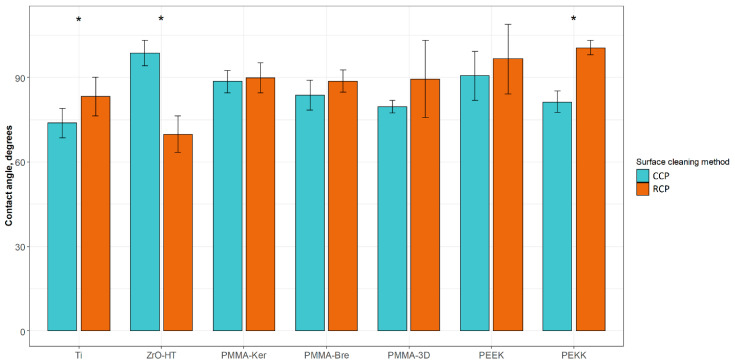
Sample contact angles using different surface cleaning protocols. The results are presented as averages +/− standard deviations. *—statistically significant differences (*t*-test, *p* < 0.05) comparing means of CCP and RCP for each material group. CCP—Conventional cleaning protocol; RCP—Research cleaning protocol.

**Figure 4:**
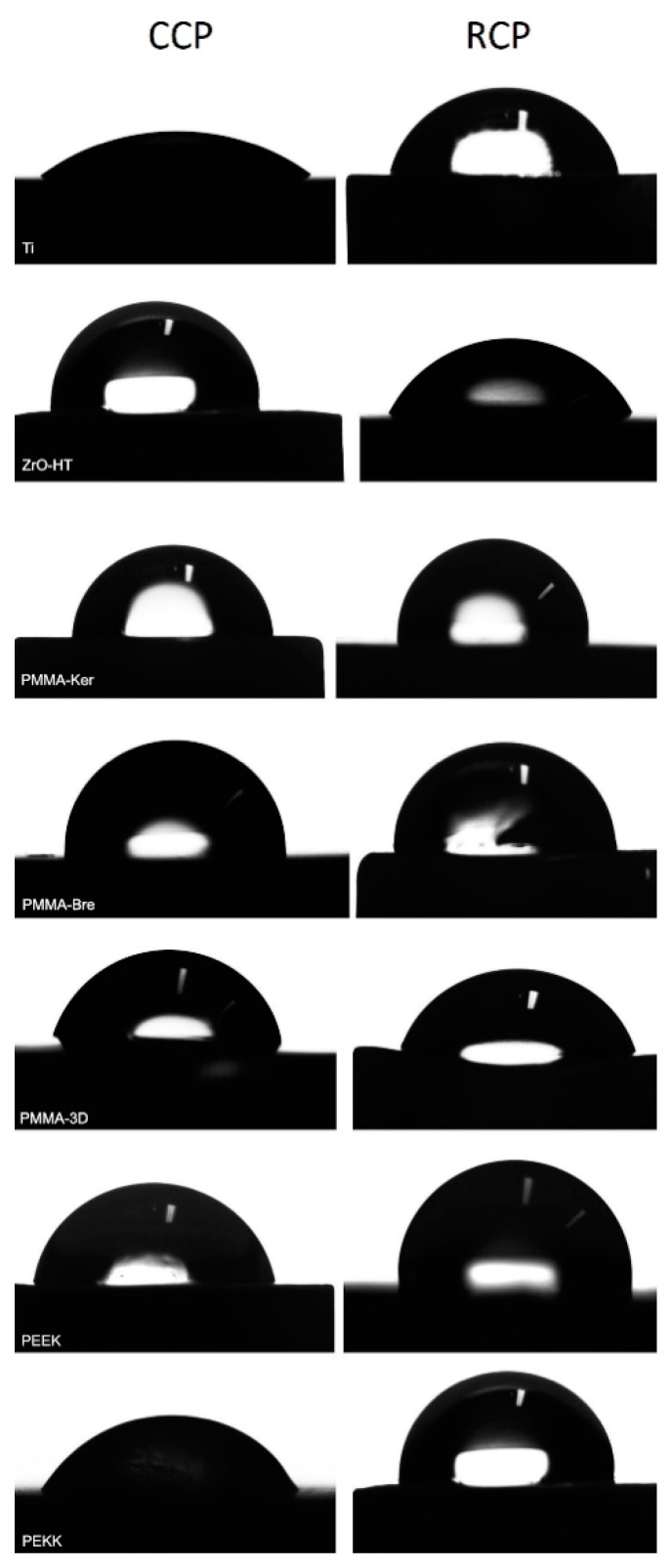
Water droplet on material surfaces used for contact angle measurement. CCP—Conventional cleaning protocol; RCP—Research cleaning protocol.

Results of water contact angle (WCA) measurements are presented in Table 4. In case of CCP, ZrO-HT group showed significantly higher (ANOVA and post hoc Tukey’s Contrasts) contact angle compared to Ti (*p* < 0.001), PMMA-Bre (*p* = 0.00163), PMMA-3D (*p* < 0.001), and PEKK (*p* < 0.001) groups. Morever, the Ti group showed a significantly lower contact angle compared to PMMA-Ker (*p* = 0.00181) and PEEK (*p* < 0.001) groups. Finally, the PMMA-3D group contact angle was significantly lower than the PEEK group (*p* = 0.03175). In case of surface preparation using the RCP, ZrO-HT group showed significantly lower contact angle compared to PMMA-Ker (*p* = 0.0107), PMMA-Bre (*p* = 0.0183), PMMA-3D (*p* = 0.0131), PEEK (*p* < 0.001), and PEKK (*p* < 0.001) groups. Furthermore, in the Ti group, the contact angle was significantly lower than the PEKK group (*p* = 0.036). Significant contact angle differences for each material group comparing two surface cleaning protocols are presented in Figure 3. Images of water droplets used for contact angle measurement on different surfaces are presented in Figure 4.

### 3.3. HGF Proliferation

**Figure 5:**
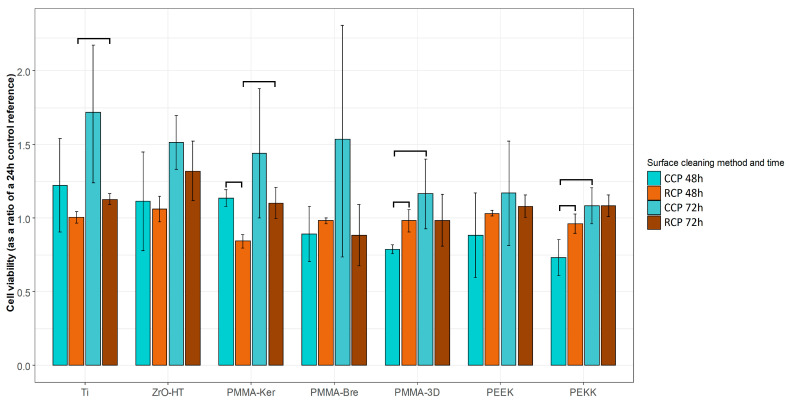
HGF proliferation on specimen surfaces after 48 and 72 h using two cleaning protocols. The results are presented as averages +/− standard deviations. Statistically significant differences (*t*-test, *p* < 0.05) between cleaning protocols and time periods are noted. CCP—Conventional cleaning protocol; RCP—Research cleaning protocol.

Cell viability data is presented in Table 5 and Table 6 for each cleaning protocol. HGF proliferation showed a tendency to increase over time for both cleaning protocols (Figure 5). Significant differences comparing both periods (*t*-test, *p* < 0.05) were detected for CCP in PMMA-3D, PEKK groups. In the case of RCP, these differences were observed in Ti, PMMA-Ker groups. In the case of the CCP, data indicates more variability in HGF proliferation. After 48 h, polymeric materials (except for PMMA-Ker) cleaned by multistep RCP showed a tendency for higher cell viability (PMMA-3D, PEKK *p* < 0.05). After 72 h, cell viability tended to be higher for conventionally cleaned materials. However, due to the high dispersion of the values, the difference between two cleaning protocols at this time point was not statistically significant.

## 4. Discussion

This study has evaluated the effect of two cleaning protocols applied to five types of polymeric materials. The surface roughness, hydrophilicity, and fibroblast cell culture response were analyzed. In clinical practice, polymers are used as temporary or permanent prosthetic materials [38,39]. Temporary abutments are an important part of the treatment course as they form and condition soft tissues during the sensitive initial healing phase [40]. Moreover, in some cases of immediate implant placement or surgical soft tissue management procedures (especially in the esthetic area), temporary abutments might serve for the entire healing period, which could last from weeks up to a few months [41,42,43]. With increase applications of one-stage surgery and immediate or early loading, permanent prosthetic materials can also be used during the healing phase [44,45,46]. The ultimate attempt during these early healing stages is to guide the cellular response of soft tissues to form the architecture and sealing around dental implant abutment similar to that around healthy natural dentition. 

The aim of this in vitro study was to evaluate the effect of five different polymer-based materials and their surface characteristics using two different cleaning protocols on HGF proliferation. The results revealed significant differences in surface roughness, water contact angle, and HGF proliferation between the groups. Therefore, the null hypothesis was rejected.

Previous findings indicated that a threshold of 0.2 µm average roughness value reduces bacterial adhesion significantly [26,47,48,49]. In this study, the roughness values of tested polymeric materials after CCP tended to be higher than the threshold, but lower after RCP. Recent research has indicated that titanium surfaces smoother than 0.1 µm might have a negative impact on fibroblast function [50]. Surface roughness in the range of 0.1–0.2 µm showed higher fibroblast adhesion than smoother or rougher surfaces for titanium, zirconium dioxide, and lithium disilicate materials [51]. In the case of CCP, only Ti and ZrO were in this range, while after RCP, all tested materials except for ZrO (0.079 +/− 0.017 µm) were in this range.

There is limited data regarding the influence of the cleaning method applied to the surface roughness parameters. Heimer et al. found that an ultrasonic bath for 6.3 min resulted in similar material surface roughness (Ra) values (0.033 µm for PEEK and 0.066 µm for PMMA) compared to other laboratory cleaning systems operating for 15–20 min [19]. The results contradict our findings, most likely due to differences in study design and methods used. In the present study, polishing protocols recommended by manufacturers were applied to each material. Moreover, in the case of CCP ultrasonic bath was used for 3 min, and finally, a non-contact surface profilometry method was used to evaluate roughness (Sa). 

Average surface roughness values (Ra/Sa) in different studies range widely: PMMA 0.02–6.2 µm [18,19,20,21,52], 3D printed PMMA (SLA) 0.39–2.97 µm [52,53], PEEK 0.032–2.52 µm [15,18,19,20,24,52,54,55], and PEKK 0.24–3.11 µm [56,57]. In some of these studies, cleaning protocol is either not stated or not used after polishing [24,52,53]. Most commonly, an ultrasonic bath filled either with water or alcohol (e.g., 70% ethanol or isopropanol) with consecutive water washing was used from 3 up to 20 min [15,18,20,21,54,55,57]. There is a high degree of heterogeneity in methodology: material specifications (inorganic filler content), surface finishing protocols, and significant variation of profilometry methods [15,18,20,21,24,52,53,54,55,56,57]. The results of the current study after both cleaning protocols are in the reported range for PMMA and PEEK but are lower for 3D printed PMMA and PEKK. In other studies, PMMA was not polished after 3D printing, and its surface roughness varied depending on object orientation during 3D printing [53,58]. Meanwhile, PEKK abutment surfaces were roughened, and bonding strength was tested [56,57]. Finally, surface profilometry method (contact vs. non-contact) and parameters (stylus tip radius, cut-off value, a field of view, measurements per surface taken, etc.) should be considered when evaluating research data [59,60,61,62].

Hydrophilic surfaces are known to be favorable for eucaryotic cells [30,31]. The hydrophobicity of the implant abutment surface is also reported to influence the adhesion of certain bacteria [63]. Wassmann et al. conducted an experiment demonstrating that hydrophobic surfaces are more attractive to *Staphylococcus epidermidis*, which causes a cytotoxic effect on human fibroblasts and therefore interferes with osseointegration and soft tissue healing [64,65,66]. Using CCP lead to hydrophobic surfaces of ZrO and PEEK, while in the case of RCP, only polymeric materials surfaces (PMMA, PEEK, and PEKK) were hydrophobic.

A tendency for increased contact angle after application of RCP was observed in all groups except for ZrO, with significant differences in Ti and PEKK groups. Changes in the surface hydrophilicity after using different cleaning protocols were also demonstrated in previous studies: ultrasonic bath alone resulted in the least hydrophilic surfaces for PMMA and PEEK compared to other laboratory cleaning methods [19].

Different water contact angle measurements were reported by other studies: PMMA 72–99° [20,21], 3D printed PMMA (SLA) 71–79° [58], PEEK 10–114° [15,20,24,54], and PEKK 64–83° [57]. The findings of the current study for PMMA and PEEK are similar to those mentioned above, but PMMA-3D and PEKK showed higher contact angles after RCP. This research showed a tendency of a more thorough cleaning method (RCP) to result in a higher water contact angle for tested materials compared to CCP.

The CCP resulted in higher variability of cell proliferation and was less predictable in the outcome. Gheisarifar et al., (2020) used ultrasonic cleaning of specimens and concluded that PEEK plasma treatment (Sa 0.68–2.14 µm, WCA 10–12°) increased HGF proliferation [15]. The present study has demonstrated that the ultrasonic cleaning based method (CCP) of PEEK surface (Sa 0.28 ± 0.1, WCA 91 ± 9°) tends to be less favorable for HGF proliferation compared to RCP (Sa 0.17 ± 0.06 µm, WCA 97 ± 12°). This tendency was not evident at 72 h. Similar results were observed with PMMA-3D and PEKK materials. PMMA-Ker demonstrated opposite outcomes in terms of HGF proliferation, favoring CCP at 48 h. Methacrylate polymers were shown to have different HGF cytotoxicity and attachment properties, depending on their composition and fabrication method [21]. This study showed similar results comparing fibroblast proliferation over time under different cleaning protocols on PMMA-Ker, PMMA-Bre, and PMMA-3D surfaces.

Another study evaluated immortalized human gingival epithelial keratinocytes (iHGEK) behavior on smooth Ti, rough ZrO, and medium PEEK surfaces and concluded similar cellular responses to all three materials [24]. This research provides similar results with HGF culture, as there were no significant differences in terms of cell proliferation between Ti, ZrO, and PEEK groups.

Though the average proliferation values tended to be higher for the CCP after 72 h, due to high variability, these differences were not significant in any of the groups (including both positive controls). This study backs the importance of multiple repetitions (at least three independent experiments) of cell proliferation experiments to evaluate the effect of the material surface. 

Furthermore, limitations of the study design must be taken into account as it was tested under the sterile, well-controlled conditions with HGF monoculture. Under clinical conditions, immediately after the placement of implant abutment, its surface becomes a subject of fibroblasts, epithelial cells, and microbial adhesion, as well as inflammatory tissue reaction. Human histology research shows that long epithelial junction predominates in contact with the transmucosal implant component [12]. Furthermore, the collagen fibers tend to be oriented parallel to the surface [12]. This can be influenced by many factors, including the implant-abutment connection, microgap, and “micro-trauma” due to connections and disconnections of the implant transmucosal component during the treatment [12]. The design of the current study did not allow to evaluate these circumstances. As results can be influenced by different types of materials, polishing and cleaning protocols, cell and bacteria types used in the studies, further research is needed to provide clinical recommendations. 

## 5. Conclusions

Considering the limitations of the current study, the following conclusions can be drawn:

- Polymer-based material surface cleaning protocol can significantly influence roughness, contact angle, and fibroblast proliferation of polymer-based materials;
- Lower surface roughness (Ra < 0.2 µm) resulted using an RCP and was higher (Ra > 0.2 µm) when the CCP was applied;
- RCP showed a tendency to reduce hydrophilicity of polymer-based material surfaces;
- CCP resulted in more variability in surface characteristics, and the cellular response was less predictable. RCP significantly favored HGF proliferation on PMMA-3D and PEKK surfaces after 48 h.

## Figures and Tables

**Table 1:** Materials used in the study.

Abbreviation	Material	Brand Name	Manufacturer
Ti	Titanium, commercially pure, grade 4	CopraTi-4	Whitepeaks Dental Solutions GmbH & Co. KG, Wesel, Germany
ZrO-HT	Zirconium oxide ceramic (3 mol% yttria-stabilized tetragonal zirconia polycrystal)	KATANA™ Zirconia HT12	Kuraray Noritake, Tokyo, Japan
PMMA-Ker	Polymethylmethacrylate	E4K PMMA Premia	Kerox Dental Ltd., St Sóskút, Hungary
PMMA-Bre	Polymethylmethacrylate composite with ceramic fillers	breCAM.multiCOM	Bredent, GmbH & Co KG, Senden, Germany
PMMA-3D	Polymethylmethacrylate (3D printed from methacrylic oligomers)	NextDent™ Crown and Bridge (C&B)	NextDent B.V., Soesterberg, The Netherlands
PEEK	Polyetheretherketone reinforced with ceramic filler	BioHPP^®^	Bredent, GmbH & Co KG, Senden, Germany
PEKK	Polyetherketoneketone reinforced with titanium dioxide	Pekkton^®^ ivory	Cendres and Métaux, Biel/Bienne, Switzerland

**Table 2:** The polishing protocols used for each material surface.

Material	Polishing Protocol per Each Surface			
Ti	EVE (R22 Item No.: 1000) White polisher 7000–10,000 min^−1^/ 30 s EVE Ernst Vetter GmbH, Keltern, Germany	EVE (CRP-R22m) Dark blue polisher 8000–15,000 min^−1^/ 30 s EVE Ernst Vetter GmbH, Keltern, Germany	Zircopol polishing paste and narrow brush 10,000 min^−1^/30 s Feguramed GmbH, Buchen, Germany	
ZrO-HT	MPF Zmax disc (Item No. 120-0001 Zmax Large Disc 22 × 4.5 mm) 5000–10,000 min^−1^/ 30 s MPF Brush Co., Nicosia, Cyprus	Edenta (R1530HP) StarGloss pink polisher for ceramics 5000 min^−1^/ 30 s EDENTA AG, Au/St. Gallen, Switzerland	Edenta (R1540HP) StarGloss green polisher for ceramics 5000 min^−1^/ 30 s EDENTA AG, Au/St. Gallen, Switzerland	Zircopol polishing paste and narrow brush 10,000 min^−1^/ 30 s Feguramed GmbH, Buchen, Germany
PMMA-Ker, PMMA-Bre	BREDENT acrylic polisher medium grey (REF P243HM10) 10,000–15,000 min^−1^/ 30 s Bredent medical GmbH & Co.KG, Senden, Germany	BREDENT Pumice polishing paste and narrow brush 5000–10,000 min^−1^/ 30 s Bredent medical GmbH & Co.KG, Senden, Germany	SILADENT TEK-1 POL Diamond polishing paste and cotton brush 10,000 min^−1^/ 30 s Siladent Dr. Böhme & Schöps GmbH, Goslar, Germany	
PMMA-3D	BREDENT acrylic polisher medium grey (REF P243HM10) 10,000–15,000 min^−1^/ 30 s Bredent medical GmbH & Co.KG, Senden, Germany	BREDENT Pumice polishing paste and narrow brush 5000–10,000 min^−1^/ 30 s Bredent medical GmbH & Co.KG, Senden, Germany	SILADENT TEK-1 POL Diamond polishing paste and cotton brush 10,000 min^−1^/ 30 s Siladent Dr. Böhme & Schöps GmbH, Goslar, Germany	
PEEK, PEKK	BREDENT acrylic polisher medium grey (REF P243HM10) 10,000–15,000 min^−1^/ 30 s Bredent medical GmbH & Co.KG, Senden, Germany	Zircopol polishing paste and narrow brush 10,000 min^−1^/ 30 s Feguramed GmbH, Buchen, Germany		

**Table 3:**
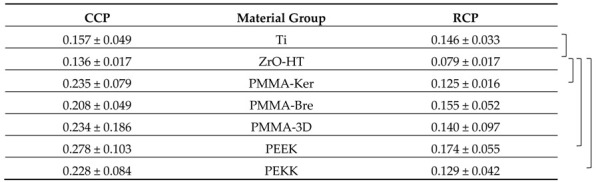
Surface roughness (Sa) average values for each material group and cleaning protocol presented in micrometers (µm) as averages +/− standard deviations.

Statistically significant (Kruskal–Wallis and pairwise Wilcoxon, *p*-adjusted < 0.05) differences comparing material group means within each cleaning protocol are marked with a bracketed notation on the respective side of the table. CCP—Conventional cleaning protocol; RCP—Research cleaning protocol.

**Table 4:**
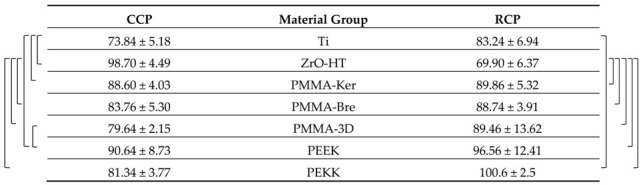
Water contact angle (degrees) average values for each material group and cleaning protocol presented as averages +/− standard deviations.

Statistically significant (ANOVA and post hoc Tukey’s Contrasts, *p* < 0.05) differences comparing material group means within each cleaning protocol are marked with a bracketed notation on the respective side of the figure. CCP—Conventional cleaning protocol; RCP—Research cleaning protocol.

**Table 5:**
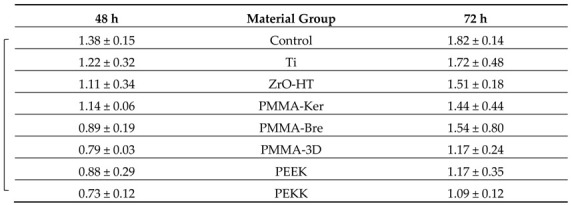
Human gingival fibroblasts (HGF) proliferation on specimen surfaces after Conventional cleaning protocol (CCP) is presented as cell viability (as a ratio of 24 h control reference).

The results are presented as averages +/− standard deviations. Statistically significant (ANOVA and post hoc Tukey’s Contrasts, *p* < 0.05) differences comparing material group means within each time period are marked with a bracketed notation on the respective side of the figure.

**Table 6:**
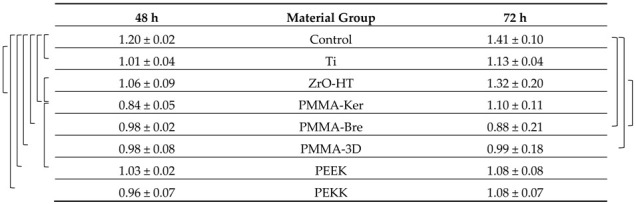
HGF proliferation on specimen surfaces after Research cleaning protocol (RCP) is presented as cell viability (as a ratio of 24 h control reference).

The results are presented as averages +/− standard deviations. Statistically significant (ANOVA and post hoc Tukey’s Contrasts, *p* < 0.05) differences comparing material group means within each time period are marked with a bracketed notation on the respective side of the figure.
